# COVID-19 Policy Response Analysis: A Canadian Perspective

**DOI:** 10.3390/ijerph21060787

**Published:** 2024-06-17

**Authors:** Andrew Klein, Mehmet A. Begen

**Affiliations:** Ivey Business School, Western University, London, ON N6G 0N1, Canada; aklein.msc2021@ivey.ca

**Keywords:** COVID-19 policies, economic and social implications, trade-offs, continuous improvement

## Abstract

The COVID-19 pandemic highlighted the challenges that go into effective policymaking. Facing a public health crisis of epic proportion, government bodies across the world sought to manage the spread of infectious disease and healthcare-system overwhelm in the face of historic economic instability and social unrest. Recognizing that COVID-19 debates and research are still actively ongoing, this paper aims to objectively compare COVID-19 responses from countries across the world that exhibit similar economic and political models to Canada, identify notable failures, successes, and key takeaways to inform future-state pandemic preparedness.

## 1. Introduction

Governing during normal times is difficult enough as is, but governing during a public health crisis becomes infinitely more challenging; satisfying all facets of society becomes a near-impossible task. Most government bodies recognized the quintessential trade-offs that existed between public safety (i.e., public health and containment of the viral spread) and both economic productivity and mental health, but governments across the world responded to those trade-offs differently. Some countries imposed stringent and restrictive measures to minimize impact on the healthcare system while others quickly re-opened to resume normal activities and minimize the economic impact, understanding that government mandates to manage the viral spread come at the cost of business closures and social isolation—each of which have profound downstream implications.

Every policy decision is filled with uncertainty, potential liability, and criticism from different groups [1]. Reflecting back on the last three years of the pandemic, it is clear that not all decisions made in the early months were the correct decisions. Although medical experts were trying to make the best decisions they could with the data and information that were available to them at the time, they were also influenced by governments and policy makers. Recognizing the challenges in the early stages in the pandemic, most notably the lack of historical data and novelty of the coronavirus, policymakers were faced with impossible decisions to make against a backdrop of resistance from all angles. This paper does not aim to criticize, but rather to apply as much objectivity to extract key insights to inform future-state pandemic preparedness.

## 2. Commentary

Comparing how different countries responded to COVID-19 presents a unique challenge. Take, for example, Canada, a parliamentary democracy, and United Arab Emirates, an absolute monarchy. Considering the fact that these two countries have fundamentally different political systems, it would not make for a fair comparison. Centralized authoritative power can manifest itself differently from a system in which the power of the prime minister is constrained by ministers and cabinet members. For that reason, we have aligned with the framework outlined by Razak and colleagues in which they identified three parameters to determine comparator countries: similar economic and political systems, per-capita income levels, and population size [2]. These three criteria enable fair comparisons between Canada, Belgium, France, Germany, Italy, Japan, Netherlands, Sweden, Switzerland, the United Kingdom, and the United States [2]. Despite creating a reasonable framework for comparison, there is no such thing as a perfect comparison; it is still critical to consider the cultural differences between these countries. Take Japan for example. A collectivistic society and the world’s most aged country (27.7% of the population are adults between the ages of 60–79 and 8.5% of the population are over the age of 80), it is likely that lower case counts can be attributed to differences in social and cultural norms. Compared to the United States, Japan exhibits fundamentally different attitudes on individualism, culture, and personal relationships [3]. Even before the pandemic, it was integral in Japanese culture to wear masks and not speak on the phone aloud in public, two variables strongly correlated with viral transmission [4]. Measures of obesity were also statistically significant. Japan’s low obesity rate is also thought to have played a role; COVID-19 death rates are 10 times higher in countries where more than half of the adult population is considered overweight [5].

**Figure 1:**
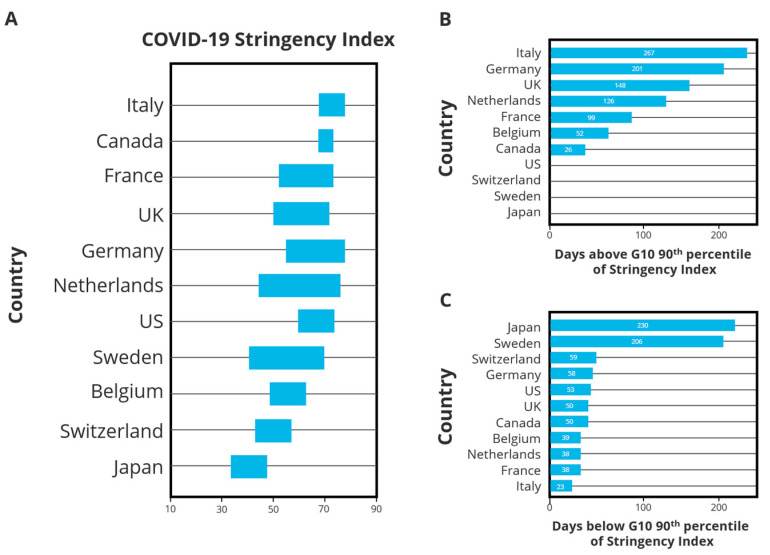
Oxford stringency index: a composite measure encompassing nine response indicators including travel bans, school closures, and workplace lockdowns, scaled from 0–100, 100 reflecting the strictest), referencing Figure 1 in [2], Canada’s response to the initial 2 years of the COVID-19 pandemic: a comparison with peer countries, Fahad Razak et al. (**A**) gives an overall stringency index for each country whereas (**B**,**C**) show number of days above and below the 90th percentile mark for each country, respectively.

One of Canada’s unique characteristics is its healthcare system. Canada’s system is publicly funded, and it offers universal coverage for necessary treatments [6]. Although there is federal oversight at the highest level, each province and territory can implement their own public health measures. Stringency index [7], a measure of actions taken by governments in response to COVID-19, is comprised of nine metrics: school closures, workplace closures, cancellation of public events, restrictions on public gatherings, closures of public transit, stay-at-home requirements, public information campaigns, restrictions on internal movements, and international travel controls (https://ourworldindata.org/covid-stringency-index, accessed on 1 March 2024). Looking at the country as a whole, Canada exhibited some of the most stringent public health measures such as long duration of school closures, prolonged workplace closures, and severe restrictions on social gatherings and international travel (Figure 1) [2]. Figure 1, reprinted from [2], depicts box plots of stringency index for each country we are comparing—days above 90th percentile of stringency index and days below 10th percentile of stringency index. From the figure, we see that Italy has the greatest number of days above 90th percentile and the lowest number of days below the 10th percentile. On the other hand, Japan and Sweden have highest number of days below the 10th percentile, indicating they are both low-stringency-index countries.

One policy that is widely recognized to be the most controversial, subject to the harshest criticism, is the vaccine mandate. Canada mandated proof of vaccination for dining in restaurants, working out at gyms, and travel and extended the mandate to truckers crossing the US–Canada border. For citizens, feeling a growing infringement upon their civil liberties, it was the vaccine mandate for truck drivers that tipped the scales. The Freedom Convoy consisted of a collection of truck-drivers, conservative groups, and independent supporters who took to the streets of Ottawa to demonstrate around parliament protesting their opposition to government overreach. Based on their response, the Canadian government only escalated the matter further by using emergency authorization to freeze bank accounts of protestors, recognized as yet another overreach of federal authorities [8].

While most countries imposed more stringent measures, Sweden was an outlier. Businesses remained open, restaurants remained open, and schools stayed open for kids under 16 [9]. In Sweden, upper secondary schools (grades 10–12) transitioned online, while lower secondary schools (grades 7–9) remained open continuing in-person education as normal. Analysing the effects of school closures, parents of lower secondary students experienced a slight increase in infection rate and lower-secondary teacher’s risk of infection doubled compared to other teacher groups [10]. Distinct from their Nordic neighbours, Sweden opposed mandating behaviours and advocated for voluntary measures, which in turn empowered citizens, increased trust, and enhanced faith in the social welfare system leading to better recommendation adherence [11].

Was it the correct decision to keep schools open? There is no simple answer, and it is still a matter that is widely debated, but it is critical to consider the public health benefit of keeping schools closed in the context of the negative impacts of virtual learning on childhood development and mental health. Though it is unclear how these findings may translate across settings, Sweden was unique in their relaxed approach to COVID-19 [12].

Excess mortality rate takes into consideration regional and national differences and encompasses all deaths both directly attributed to COVID-19 and indirectly (e.g., deaths of despair by suicide, death due to hospital avoidance). This metric is beyond conventional metrics like infection rate and mortality rate, and it introduces greater objectivity to reflect the pandemic’s wide-spread impact. In addition to Sweden, other Nordic countries like Finland and Norway had the lowest average lockdown rate and also experienced the lowest excess mortality as minimized economic costs [13]. Though subject to excessive criticism, Sweden adequately assessed the risk levels by age and managed to protect the elderly more effectively than most, recognizing that the virus was not overly dangerous to young people [9]. Relative to other G10 countries, Sweden experienced zero excess mortality among individuals over 75; lower than the US (highest excess mortality), the UK, France, Belgium, Switzerland, the Netherlands, and even Germany, which instituted strict lockdown measures [14].

Analysing the Oxford Stringency Index (Figure 1), which measures variation in stringent protocols over the course of the pandemic, Canada finished with the second highest score at 70.8 [2]. Relative to other G10 countries, Canada had the second lowest infection rate, only ahead of Japan, at 82,700 cases per million [2]. All other countries were above 100,000 cases per million residents. Japan, the country with the lowest infection rate at 27,600 per million, exhibited the lowest stringency index. These findings demonstrate an inconclusive relationship between stringent protocols and infection rate, further complicating our understanding.

Despite recording five million cases and only just over 23,000 deaths, Japan’s response was not all successful. The healthcare system was stretched too thin with too few beds to accommodate both non-COVID-19 and worsening COVID-19 illnesses [4]. Vaccine roll-out across the country was chaotic, and the arrival of the Olympics in 2021 brought the Delta variant along with it [4].

At the outset of the COVID-19 pandemic, conflict between US government agencies led to incoherent communication coming out of Washington. When clear messaging and a united front was most essential, the divided USA government failed to quell the fears and manage the crisis, leading to increased hostility between political groups, more partisan division, and social disorder [15].

At the outset, the CDC (Centres for Disease Control and Prevention) and WHO (World Health Organization) both stated that face masks were not necessary in efforts to retain the supply for essential healthcare workers. President Trump downplayed the threat, likened it to a run-of-the-mill virus, and contradicted the altered narrative put forward by the CDC expressing that “disruptions to daily life could be severe” [15]. Incongruencies between the White House and top public health experts made communication confusing and challenging. Mixed messages resulted in a loss of faith in the USA institutions—Americans lost faith in scientists, public health, the media, and elected officials [16].

Whether or not you wore a mask signified your political alignment. The US President openly mocked those who wore a mask, and many people in the US internalized the philosophy that wearing a mask infringed on their constitutional rights and individual freedoms. Many groups experienced growing scepticism around the intentions of public health to act in the public’s best interest. Therefore, masks became the ultimate symbol of controversy and one of the leading drivers behind the newly inflamed culture wars.

Beyond their many failures, major developments in scientific research and genome sequencing enabled a record setting timeline for the development of COVID-19 vaccines [17]. Vaccine development usually takes upwards of 5 years, but COVID-19 vaccines were developed, tested, and authorized for use within the span of 11 months [18]. Overall, through Operation Warp Speed, the US was highly efficient in developing a vaccine that was approximately 95% effective in preventing symptomatic COVID, not preventing transmission—but that was another notable failure in communication surrounding vaccine messaging [19]. As much as vaccine development was a resounding success, the same cannot be said for the vaccine roll out. The fragmented and disjointed response can largely be attributed to the already overburdened and underfunded states and public health authorities managing their respective crises [20].

The United Kingdom failed in their early optimism to achieve herd immunity by infection [21]. By failing to take aggressive actions early on and delaying lockdown protocols, England faced exceedingly high death rates [21]. In light of those failures, the UK was first to deploy vaccines as well as vaccinate and supply boosters for 50% of their population [22]. One success was the UK’s methodical, deliberate, and vaccine efficient strategy that was proved successful and protected the most vulnerable segments of the population [22]. Due to the successes with the vaccine program, the UK government was able to ease restrictions earlier than other countries during the spring of 2021 [22].

Of all G10 countries, Italy had the highest stringency level over the course of the pandemic and yet also had the highest mortality rate per million citizens. This, again, only serves to further complicate our understanding of the relationship between stringent measures (e.g., school closures, travel bans, lockdowns) and mortality risk [2]. Within the first few months of the pandemic, Italy was more reactive than proactive, gradually increasing restrictions until they were applied to the entire country [23]. Italy’s reactive approach set them behind the curve from the outset to the point where it became impossible to get ahead of the exponential viral spread.

Lombardy, a region in northern Italy, has a population of roughly 10 million people, one sixth of the Italian population, and yet accounted for 37% of cases and 53% of deaths in the country [24]. Lombardy had a death rate six times higher than the rest of the country [24]. Despite the greater population density, public health decisions in Lombardy enabled private and public health systems to compete for funding. Inevitably, this led to investment in more lucrative spaces and reduced funding from beds in the public system [25]. These decisions set Lombardy on a worse trajectory compared to other regions in the country. Veneto, a region in Italy’s northeast took a much more proactive approach compared to Lombardy. Rigorous testing, proactive contact tracing, and special efforts to protect essential workers resulted in positive health outcomes and a reduced burden on hospitals [23].

## 3. Discussion

School closures were a widely controversial topic. Amid all the confusion and uncertainty, one thing was overwhelmingly certain; students at home, subject to inappropriate learning environments, isolated from their peers during critical stages of development, unmotivated, and distracted, were learning less at home during a lockdown compared to any given year [26]. Teachers struggled to adapt to online-based instruction solutions, there was a drastic drop in coursework completed by students and increased dispersion among test scores [10].

School closures served to exacerbate income inequality. Of all the variables measured to analyse the influence of student’s performance studying online, including sex, school grade, subject, or prior performance, home environment had the greatest influence [10]. Learning losses were most prevalent among students from disadvantaged homes, especially when parental education level was lowest [10]. With schools closed, the home evolved to become a place to live, a place to work, and a place to learn, thereby blurring the boundaries between different roles and confusing familial relationships [27]. Students forced to learn online further complicated matters for parents who were mandated to return to work in person (i.e., essential workers).

From a socioeconomic standpoint, the vast majority of policies implemented in North America benefited the upper class and failed to protect the low-income segments of the population. Remote work gave rise to the laptop class. As a collection of largely affluent and educated individuals, this group of workers transitioned online seamlessly with virtually no risk to their health or income [28]. Though the virus affects everyone, it affects everyone differently and to different degrees. An individual’s COVID-19 experience is influenced primarily by age, access to healthcare, the ability to work from home, the need to educate children remotely, and the extent to which income was interrupted, only further exacerbating the growing economic divide [29]. As COVID-19 ravaged much of the world, ultra-wealthy individuals watched their wealth grow drastically; billionaires across the world increased their fortunes by 54%, a collective increase from roughly $8 trillion to more than $12 trillion [30]. With record low interest rates and prospering portfolios, many high-income earners sought to purchase additional rural properties to satisfy their desires to work-from-home outside the dense urban city. Individuals living in low-income counties in the USA were 67% more likely to die from the respiratory virus [28]. Infections were concentrated mainly among low-paid workers who did not have the luxury of remote work, were unable to access paid sick leave, and whose working conditions made social distancing challenging.

Beyond the economic implications [31], COVID-19 had detrimental effects on mental-health [32,33]. There is ample evidence on the relationship between social connectedness and health; individuals who have meaningful and regular social exchanges, a sense of community support, and strong bonds with others are more likely to have better health outcomes [34]. Furthermore, they are better able to cope during times of increased stress [34]. COVID-19 capitalized on these two fronts; not only was the virus an incredibly stressful life event but it also directly impaired our social connectedness by way of social restrictions. Increased restrictions led to feelings of loneliness and social isolation, perpetuating worse health outcomes [35].

Over the course of the pandemic, there were marked increases in anxiety, depression, as well as alcohol and substance abuse [36]. Fears surrounding the virus, potential long-term complications, in tandem with increasing financial burdens further compounded by a diminished sense of community support resulted in deaths of despair, deaths indirectly attributed to COVID-19 by suicide, drug overdose, and alcohol abuse. From April 2020 to April 2021, the USA reported a 28.5% increase in drug overdose deaths [37].

Overwhelmed by COVID-19 cases, hospitals in Ontario paused non-urgent surgeries and suspended cancer screening to increase resource capacity for COVID-19 patients [38]. In Ontario alone, 951,000 fewer screening tests were administered in the first year of the pandemic compared to 2019 [39]. Beyond cancelling surgeries, the pandemic had tremendous impact on cancer screening. Across Canada, there have been 560,000 fewer surgeries during 16 months of the pandemic compared to 2019 [40]. During the first year of COVID-19, Ontario experienced roughly 40% fewer mammograms and pap tests, which detect breast cancer and cervical cancer, respectively [41]. One study has shown that a four-week delay in cancer treatment can increase the risk of death by 10% [41]. Delays in surgeries and screening can have devastating consequences—likely indicative of thousands of cancer cases gone untreated, which will be more costly in the long-term.

Recognizing the trade-offs and far-reaching economic and social impact, one may be inclined to ask the following questions:

- at what point is the public health benefit of restrictive lockdowns, cancelling non-urgent surgeries, and allocating all hospital resources to COVID-19 patients outweighed by the resulting economic despair, social isolation and mental health costs, and the risk of delayed surgeries to other sick patients and the development of new diseases gone undetected due to decreased screening?
- at what point is the public health benefit of keeping schools closed outweighed by the negative impacts of virtual learning on childhood development and mental health?
- at what point is the public health benefit of keeping business closed outweighed by the despair experienced by many workers who have lost their livelihoods, and in most cases, their purpose?
- at what point is the public health benefit of the government overstepping to manage a pandemic outweighed by cost of loss of individual autonomy and infringement on basic civil liberties?

## 4. Insights

Over three years have passed now from the initial lockdowns, and with that we have accumulated an abundance of data from various countries that took different approaches. Recognizing these were impossible decisions to make in the early weeks, we now have the luxury of hindsight to reflect with greater objectivity to extract relevant insights, learn from them, and ultimately inform future-state pandemic preparedness and avoid costly mistakes learned from COVID-19. We learned that clear communication across government agencies is critical. Initially, the USA’s senior-most public health official and head of the National Institute of Allergy and Infectious Diseases, Dr. Anthony Fauci, stated that there would be no reason to wear a mask and that it is in fact “not providing the perfect protection that people think it [does]” [42]. In reality, this sentiment was expressed to ensure mask supply for essential healthcare workers, thereby misleading the public and ultimately sowing distrust when this information later came to light [42]. This confusion drives polarization between political groups and erodes trust in public institutions. Public trust is needed to manage a global pandemic and fuel collective cooperation [43]. Gaining trust takes a lot of effort, and losing it is all too easy. Public officials need to own up to their mistakes—transparency is key. No reasonable person can expect flawless execution in a pandemic of historic proportions, but to deny reality is to cast ignorance upon the public; after all, *experience is the name we give to our mistakes*.

We learned that a central authority or task force is critical to drive a coordinated strategy and mitigate a fragmented or disjointed response. This authority must also ensure a comprehensive pandemic preparedness plan as well as Personal Protective Equipment (PPE) stockpiles to ensure ample supply is accessible when needed. Restrictive measures around closures, testing, and tracing need be actioned early to have a meaningful impact; delayed actions are futile.

We learned that health risk factors must be assessed appropriately by age demographic; the threat of COVID to one’s life increased exponentially as they got older. Compared to individuals aged 18–29, individuals aged 65–74 were 60 times more likely to die from the virus. That risk factor more than doubled in the next decade among individuals aged 75–84 at 140 and among those older than 85, the risk of death was 360 times the younger demographic [44]. All that to say, the risk that COVID posed was significantly greater among the elderly demographic. Age and co-morbidities (e.g., heart disease, diabetes) both serve as catalysts to amplify the threat of the coronavirus; those two also tend to go hand in hand. These risk profiles must be factored into policy decisions—for example, greater focus and care around long-term care facilities, most notably, an area in which the USA failed spectacularly [45].

In developing policies, not all demographics should be pooled together as evidenced by varied risk profiles. The Great Barrington Declaration is a letter authored by public health scientists and epidemiologists and signed by physicians and scientists all over the world that advocates for a different approach to COVID-19 policies. Pointing to deteriorating mental and physical health, worsening cardiovascular disease, and the decline in cancer screenings, the authors advocate for Focused Protection; a novel approach that enables a return to normalcy for low-risk individuals and heightened protection for those who are high-risk—most notably, the elderly [46].

Our study has several limitations. We compare only a number of countries that are similar in terms of their economic and political systems, per-capita income levels, and population sizes. Other countries with different geographic and socio-economic conditions could be different [47,48,49]. Furthermore, we cannot compare the efficacy of the measures in detail country by country since data and evidence are still being investigated and debated.

## 5. Conclusions

In the face of so much uncertainty, data-driven insights to inform policy development is the ultimate pursuit—the ideal to strive for. It is important to introduce as much objectivity into subjective matters. Recognizing the trade-offs and nuances that go into policy making, one can recognize there is no one correct path, but many different paths.

Facing a pandemic of this scale and magnitude, government officials will not know how best to manage school or business closures but should look to the data and models to inform their decisions; for example, meaningfully and comprehensively analyse the risk of hospitalization or death for school-age students, mental health scores, efficacy of remote learning, and deaths of despair attributed to substance abuse.

That is the beautiful thing about data and models; they tell their own stories independent of feelings or political motives. New hypotheses should continually be introduced and tested rigorously. As new research findings are produced and the data evolve, so too should the policies put in place. Government officials should develop the necessary infrastructure, human capital, and processes so that (i) data can collected, processed, and analysed quickly; (ii) models can be developed and re-developed as needed; (iii) policy decisions can be given promptly and revised when needed. Furthermore, governments should remember to develop and revise policies so that maximum protection for those vulnerable and continuation of normal life (e.g., economic activity, kids’ schools) could be achieved. Current developments in analytics, machine learning and artificial intelligence can help to automate and fasten some of these efforts. These new technologies will be essential to develop policies, guidelines, detection, and treatment possibilities and tools. Furthermore, they will also provide improved and efficient decision making for future pandemics.

Finally, cooperation, data and information sharing, and coordination among different agencies are essential for an efficient and effective response to a pandemic. This is not only on a country level but between countries and world-wide organizations. The COVID-19 era presented some good examples of such collaboration, e.g., sharing of data, information, vaccines, and personnel. However, there are many factors that affect this cooperation and coordination [50] and there is always room for improvement.

## Data Availability

All data and article sources have been cited.
